# Editorial: Challenges and opportunities of TKIs in the treatment of NSCLC patients with uncommon mutations

**DOI:** 10.3389/fonc.2022.970315

**Published:** 2022-07-26

**Authors:** Chunxia Su, Shi-Yong Sun

**Affiliations:** ^1^Department of Oncology, Shanghai Pulmonary Hospital & Thoracic Cancer Institute, Tongji University School of Medicine, Shanghai, China; ^2^Department of Hematology and Medical Oncology, Emory University School of Medicine and Winship Cancer Institute, Atlanta, GA, United States

**Keywords:** non-small cell lung cancer, uncommon mutation, tyrosine kinase inhibitor, targeted therapy, immunotherapy, resistance

Substantial advances have been made in our understanding of the molecular biology of cancer, leading to profound progression in the fields of diagnosis and treatment of non-small cell lung cancer (NSCLC). More recently, many uncommon mutations are gaining attention in NSCLC, including epidermal growth factor receptor exon 20 insertion (EGFR 20ins), anaplastic lymphoma kinase (ALK), human epidermal growth factor receptor 2 (HER2), rearranged during transfection (RET), v-Raf murine sarcoma viral oncogene homolog B (BRAF), neurotrophic tropomyosin receptor kinase (NTRK), and others (1). Pharmaceutical agents developed to target classical driver mutations detected in NSCLC patients have provided profound and durable responses compared with conventional chemotherapy. This paradigm shift can also be seen in the realm of uncommon mutations. Ongoing studies and clinical trials of tyrosine kinase inhibitors (TKIs) for uncommon targets are gaining insights into possible treatment options to enable long-term survival of patients. Since immunotherapy serves as the backbone treatment in mutation-negative populations, the efficacy of immunotherapy or immunotherapy-based combinations has also been evaluated in patients harboring uncommon targets. Furthermore, other novel therapies and platforms have been developed and tested to provide more possibilities and options for these patients.

In this special issue, we compiled a series of papers including original research, reviews and case reports that focus on recent advances and challenges in the treatment of lung cancer patients with uncommon driver mutations. For rare EGFR mutations, Xu et al. reported that two patient-derived xenografts in zebrafish embryos from two patients harboring EGFR 20ins received precision treatment. Zebrafish were inoculated with tumor cells and cultured in osimertinib-containing medium to predict a clinical response. Their study demonstrated the applicability of zebrafish models for testing targeted drugs. Yang et al. identified five metastatic NSCLC patients with EGFR p.L747P mutation and found that afatinib achieved numerically longer progression-free survival (PFS). Dynamic simulations and *in vivo* experiments demonstrated that afatinib had the best binding affinity and significantly inhibited p.L747P-mutant tumor growth. Feng et al. reported three locally advanced NSCLC patients with EGFR sensitive mutations switching to aumolertinib, a novel third generation EGFR-TKI, as neoadjuvant therapy after 1-2 cycles of preoperative chemotherapy neoadjuvant therapy. Excellent tumor remission and downstaging were achieved to allow surgical treatment, and no tumor recurrence was observed until the latest follow-up. This may indicate that aumolertinib was clinically applicable and could be a viable option in the neoadjuvant phase of therapy.

Nowadays, ALK-altered NSCLC can be treated with a variety of effective ALK inhibitors and a number of next-generation ALK-TKIs have already been developed. Peng et al. reviewed recent studies and summarized the efficacies and safety profiles of ALK-TKIs and other therapies with data from preclinical and clinical trials. They also proposed several key points regarding treatment sequencing strategies, resistance mechanisms of ALK-TKIs and toxicity problems when giving these drugs.

HER2 aberrations are comprised of three distinct formations: mutation, amplification and overexpression. They were originally discovered in breast and gastric cancer, but their role in lung cancer is gaining increasing attention. Yu et al. reviewed available data and described the biological function of HER2 and its dysregulation in NSCLC, as well as clinical characteristics of patients. Further, they provided a comprehensive overview of traditional and emerging therapies including chemotherapy and monoantibody, non-selective TKIs, new-generation TKIs and antibody-drug conjugates (ADCs). Of note, ADC-based therapy seems to provide the best clinical outcomes among all treatment regimens, which sheds new light on the management of HER2-altered NSCLC.

There are two real-world studies concerning treatment options for RET-fusion positive NSCLC patients. Meng et al. retrospectively analyzed the characteristics and clinical outcomes of patients with RET-fusion-positive NSCLC receiving RET-TKI, multi-kinase inhibitor (MKI), chemotherapy and immunotherapy-based regimens from three centers. The results showed that RET-TKI remained the best choice for a better response rate and PFS. Chemotherapy, especially with angiogenesis inhibitors, was still a good choice, while the other two regimens should not be recommended for this patient group. Zhou et al. reported a case involving a stage IIIA lung adenocarcinoma patient harboring RET rearrangement who was treated with pralsetinib as neoadjuvant target therapy. Pralsetinib exhibited a significant response and transformed the unresectable tumor into a resectable one. Additional clinical trials are warranted to verify the effect of pralsetinib for locally advanced NSCLC.

BRAF activation consists of several distinct forms including V600 and non-V600 mutations, rearrangements, fusions, in-frame deletions and insertions. Yan et al. and Sun et al. discussed the diagnostic challenges of BRAF mutations, therapeutic strategies and post-therapeutic evolutionary pathways of BRAF, and also the mechanisms of resistance to BRAF-TKIs. NTRK fusion has become increasingly studied in lung cancer. Liu et al. provided a panoramic view of the function of NTRK genes, the diagnostic techniques for NTRK fusions, the clinical data on TRK inhibitors and their resistance mechanisms. These reviews provide us with a comprehensive view of the current landscape of BRAF and NTRK alterations in NSCLC, but there are still many unsolved issues to be addressed.

Personalized medicine has revolutionized the therapeutic landscape of lung cancer with molecular alterations in the past two decades. Since most uncommon mutations are associated with poor to moderate efficacy of immunotherapy, their identification and the development of new-generation drugs are pivotal for designing improved treatment strategies. Attempts to treat other rare mutations are being investigated in addition to those discussed above, such as MET, ROS1, FGFR, STK11, etc. The growing use of next-generation sequencing (NGS) platforms has expanded the panel of genes that can be detected and targeted, and noninvasive liquid biopsy techniques will provide more information regarding efficacy monitoring and resistance mechanisms in real-time. With novel therapies springing up, further investigation should continue to evolve and lead to improve patient outcomes.

Finally, we thank all the authors for their inspiring contributions to this Research Topic and hope that these papers will provide our readers with a deep understanding of the current status and future directions in the treatment of NSCLC patients with uncommon mutations.

## Author contributions

All authors listed have made a substantial, direct and intellectual contribution to the work, and approved it for publication.

## Funding

This study was supported by the National Natural Science Foundation of China (grant numbers: 81874036, 82072568), the Science and Technology Commission of Shanghai Municipality (grant number: 19411971100) and the Shanghai Shenkang Hospital Development Center (grant number: SHDC12020110).

## Conflict of interest

The authors declare that the research was conducted in the absence of any commercial or financial relationships that could be construed as a potential conflict of interest.

## Publisher’s note

All claims expressed in this article are solely those of the authors and do not necessarily represent those of their affiliated organizations, or those of the publisher, the editors and the reviewers. Any product that may be evaluated in this article, or claim that may be made by its manufacturer, is not guaranteed or endorsed by the publisher.
